# Coronary artery calcification score and 19 biomarkers on cardiovascular events; a 10-year follow-up DanRisk substudy^☆^

**DOI:** 10.1016/j.athplu.2024.09.003

**Published:** 2024-09-24

**Authors:** Mie Schæffer, Jeppe Holm Rasmussen, Maise Høigaard Fredgart, Selma Hasific, Frederikke Nørregaard Jakobsen, Flemming Hald Steffensen, Jess Lambrechtsen, Niels Peter Rønnow Sand, Lars Melholt Rasmussen, Axel CP. Diederichsen

**Affiliations:** a Department of Cardiology, Odense University Hospital, Denmark; b Department of Cardiology, Sygehus Lillebælt Vejle, Vejle, Denmark; c Department of Cardiology, Svendborg Hospital, Svendborg, Denmark; d Department of Cardiology, University Hospital of Southern Denmark, Esbjerg, Denmark; e Department of Regional Health Research, University of Southern Denmark, Esbjerg, Denmark; f Centre for Individualized Medicine in Arterial Diseases, Odense University Hospital, Odense, Denmark; g Department of Clinical Biochemistry and Pharmacology, Odense University Hospital, Odense, Denmark; h Cardiovascular Centre of Excellence, University of Southern Denmark, Odense, Denmark

**Keywords:** Coronary artery calcification score, European HeartScore, Biomarkers, Risk assessment, Risk prediction, Cardiovascular disease

## Abstract

**Aim:**

The SCORE2 algorithm is recommended to estimate risk of cardiovascular disease (CVD). Coronary artery calcification (CAC) score is expensive but improves the risk prediction. This study aims to determine and compare the additive value of CAC-score and 19 biomarkers in risk prediction.

**Methods:**

Traditional cardiovascular (CV) risk factors, CAC-score, and a wide range of biomarkers (including lipids, calcium-phosphate metabolism, troponin, inflammation, kidney function and ankle brachial index (ABI)) were collected from 1211 randomly selected middle-aged men and women in this multicenter prospective cohort in 2009–2010. 10-year follow-up data on CV-events were obtained via the Danish Health Registries. CV-event was defined as stroke, myocardial infarction, hospitalization for heart failure, coronary artery revascularization or death from CVD. The association between SCORE2, CAC-score, biomarkers, and CV-events was assessed using cox proportional hazard rates (HR) and compared using AUC-calculation of ROC-curves. Finally, net reclassification improvement (NRI) was calculated.

**Results:**

92 participants had CV-events. Adjusted for risk factors, CAC-score was significantly associated with events (adjusted HR 1.9 (95%CI:1.1; 3.3), 3.6 (95%CI:1.9; 6.8), and 5. (95%CI:2.6; 10.3) for CAC-score 1–99, CAC-score 100–399 and CAC-score ≥400, respectively. HR for the highest quartile of CRP was 2.3 (95%CI:1.2; 4.5), while none of the remaining biomarkers improved HR. Adjusted for SCORE2, the CAC-score improved AUC (AUC_CAC_: 0.72, AUC_SCORE2_: 0.67, *p<*0.01). A combination of selected biomarkers (total cholesterol, low-density lipoprotein, phosphate, troponin, CRP, and creatinine) borderline improved AUC (AUC_Biomarkers + SCORE2_: 0.71, AUC_SCORE2_: 0.67, *p=*0.06). NRI for CAC score was 63 % (*p<*0.0001).

**Conclusion:**

CAC-score improved prediction of CV-events, however the selected biomarkers did not.

## Teaser

This study aimed to look at a wide range of blood samples to assess if these could improve the risk assessment to the same extent as a coronary artery calcification (CAC) score, as this is known as a strong predictor of cardiovascular diseases (CVD). None of the evaluated markers were able to improve the risk assessment like CAC-score. This study confirmed CAC-score to be a strong predictor of cardiovascular events.

## 1 Introduction

Accurate risk assessment of cardiovascular disease (CVD) is essential for clinical decision making. Traditional risk stratification algorithms, like the European HeartScore (SCORE and SCORE2) [1,2] or Framingham Risk Score (FRS) [3], are population based algorithms used for calculating the individuals overall 10-year risk of fatal and non-fatal CVD [4]. Prevention of CVD is based on healthy lifestyle advice and medical treatment of hypertension and hyperlipidemia according to the calculated risk. Still, these risk scores are suboptimal for individual risk assessment as some develop CVD despite a low risk, while others at high risk remain healthy [5].

Coronary artery calcification (CAC) score is easily derived from non-contrast cardiac computer tomography (CT) scan. It is an accurate measurement of subclinical atherosclerosis and has been proven to be a reliable marker in prediction of cardiovascular (CV) events [[6], [7], [8]]. The use of CAC-score is recommended for asymptomatic adults at intermediate risk to improve risk stratification, but implementation of a CT-scan in a screening program may not be cost-effective [4].

A wide range of biomarkers could be an alternative to the traditional risk stratification, as they have the potential to increase feasibility and cost-effectiveness. Apart from lipids, there is yet no proof of the ability of biomarkers to improve the risk stratification of CVD [4].

This study aimed to assess whether a wide range of biomarkers, which included the lipid metabolism, the calcium-phosphate metabolism, troponin I, inflammation markers, markers of kidney function and ankle brachial index (ABI), would be able to improve CV risk assessment in a population without prior CVD. With 10-years of follow-up, our specific objectives were to first determine the risk prediction of CV-events for CAC-score and 19 biomarkers. Secondly, to compare the additive value of CAC-score and biomarkers to the traditional risk assessment.

## 2 Methods

### 2.1 Study population

Participants were included from the DanRisk cohort [5]. In 2009–2010, 1825 randomly selected men and women living in Southern Denmark, who were born in either 1949 or 1959, were invited to a CT-based screening examination at four regional medical centers: Odense, Vejle, Esbjerg and Svendborg. 1257 (68.9 %) accepted the screening offer. Exclusion criteria in this study were patient-reported previous CVD or CVD as specified in the Danish registers (n = 46), leaving 1211 (66.4 %) participants for this study.

### 2.2 Screening examination

The screening examination included an assessment of CV risk factors, a non-contrast CT-scan, measures of blood pressure including calculation of ABI, and blood samples.

At baseline, CV risk factors including age, sex, smoking, medical treatment, family history of CVD, body-mass index (BMI), and blood pressure (including ABI) were recorded. Hypertension was defined as systolic blood pressure ≥140 mmHg, diastolic blood pressure ≥90 mmHg or anti-hypertensive treatment. Diabetes was defined as fasting blood glucose >7 mmol/l or as treated with antidiabetic medication, self-reported. Participants were labeled with hyperlipidemia if low-density lipoprotein (LDL) ≥3 mmol/l, total cholesterol ≥5 mmol/l or if taking lipid-lowering treatment. The European HeartScore, SCORE2 [2] was calculated from a combination of risk factors and biomarkers (sex, age, systolic blood pressure, total cholesterol, high-density lipoprotein (HDL) and smoking status), and categorized by its 10-year risk assessment: low (<1 %), moderate (≥1 % < 5 %), high (≥5 % < 10 %) and very high (≥10 %). CAC-score was measured by a non-contrast CT-scan [9] and categorized as no (=0 AU), mild (1–99 AU), moderate (100–399 AU) or severe calcification (≥400 AU). The methodology for obtaining CAC-score data have previously been described [5]. Blood samples were analyzed for biomarkers, which included the lipid metabolism (triglyceride, HDL, LDL and total cholesterol), calcium-phosphate metabolism (calcium, phosphate, calcium-phosphate product (CPP), vitamin D2 and D3, parathyroid hormone (PTH) and osteoprotegerin (OPG)), troponin I, inflammation markers (C-reactive protein (CRP), soluble urokinase-type plasminogen activator receptor (suPAR)) and markers of kidney function (creatinine, estimated glomerular filtration rate (eGFR) cystatin C and urate). All biomarkers were categorized for analysis by interquartile range (IQR), apart from CRP, for which IQR were categorized by sex.

### 2.3 Follow-up data

The study includes 10 years of register-based follow-up. Follow-up data were collected through the Danish Health Data Authority via the Danish Central Patient Register (CPR-register), the Danish Register of Causes of Death (Death-register) and the Danish National Patient Register (Diagnosis-register). Dates of death or emigration and cause of death were extracted from the CPR-register and Death-register, respectively, and diagnosis codes and date of admission were extracted from the diagnosis-register. The extracted data was used to define the first CV-event, which was defined as non-fatal stroke (DI61, DI63-64), non-fatal myocardial infarction (MI) (DI21, DI23), hospitalization for heart failure (DI50), coronary artery procedure (KFN-), and death from CVD (I00-99). Participants with no event during follow-up, were given an end-date for follow-up, 10 years from their screening date.

### 2.4 Statistics

Kaplan Meier diagrams were made for SCORE2, CAC-score and biomarkers to display survival estimates for each risk category over the 10-year follow-up time. For the statistical analysis, the association between SCORE2, CAC-score, biomarkers, and time to first CV-event was assessed using the cox proportional hazard survival analysis and compared using DeLong’s area under the curve (AUC) calculation of Harrell’s C receiver operating characteristic (ROC) curves. The net reclassification improvement (NRI) for inclusion of CAC-score was also calculated. The regression models for SCORE2 were left unadjusted because of its calculation being based on CV risk factors. The regression models for CAC-score, selected biomarkers (calcium-phosphate metabolism, troponin I, inflammation markers, markers of kidney function and ABI) were adjusted for CV risk factors: sex, age, BMI, smoking status, hypertension, diabetes, hyperlipidemia, and family history of CVD. The regression model for biomarkers of the lipid metabolism was adjusted for the same CV risk factors, except hyperlipidemia, but including statin-use. The assumption of proportional hazards was tested using the Schoenfeld proportional assumptions test. The hazard ratio (HR) for SCORE2, CAC-score and each of the biomarkers were compiled and presented within a Forest Plot. AUC for CAC-score, each individual biomarker, and a combination of post-hoc selected biomarkers (total cholesterol, low-density lipoprotein, phosphate, troponin I, CRP, and creatinine), was calculated from a ROC-curve, adjusted for SCORE2. The combination of biomarkers was selected due to their proven association with CVD in previous studies. All analyses were performed using STATA/MP 17.0 (StataCorp LLC, College Station, Texas, USA).

### 2.5 Ethical considerations

This study implied no patient contact, and all data were already available. The baseline screening examinations in 2009–2010 were approved by the Regional Scientific Ethical Committee for Southern Denmark (S-20080140).

## 3 Results

### 3.1 Baseline population characteristics, SCORE2, CAC-score and biomarkers

Descriptive baseline characteristics as well as mean values, standard deviations, and IQR of each biomarker collected at baseline are presented in Table 1. This study had an even distribution of men and women, as well as an even distribution of 50- and 60-year-olds. The risk-categorization of SCORE2 resulted in 16 (1.3 %) participants with a low-risk, 816 (67.4 %) had a moderate risk, 338 (27.9 %) had a high risk and 41 (3.4 %) had a very high risk of CVD within 10 years. After categorization of CAC-score, 666 (55.1 %) participants had no calcification, 364 (30.1 %) had mild, 112 (9.3 %) had moderate and 66 (5.5 %) had severe calcification.

**Table 1:** Descriptive Baseline Characteristics from the DanRisk study population.

Age (mean (SD))		55.3	(5.0)
Age 50 (n (%))		600	(49.5 %)
Age 60 (n (%))		611	(50.45 %)
Sex (n (%))			
men		569	(47.0 %)
women		642	(53.0 %)
Body mass index (mean (SD))		27.0	(4.8)
Smoking Status (n (%))			
Never		500	(41.3 %)
Former smoker		405	(33.4 %)
Current smoker		306	(25.3 %)
Family History of CVD (n (%))		282	(23.3 %)
Hypertension (n (%))		253	(20.9 %)
Diabetics (n (%))		35	(2.9 %)
Hyperlipidemia (n (%))		937	(77.4 %)
Statin users (n (%))		142	(11.7 %)
Biomarkers			
Triglycerides, mmol/L (n, mean (SD))	1211	1.56	(1.07)
HDL, mmol/L (n, mean (SD))	1211	1.52	(0.47)
LDL, mmol/L (n, mean (SD))	1211	3.25	(0.91)
Total Cholesterol, mmol/L (n, mean (SD))	1211	5.49	(1.03)
Calcium, mmol/L (n, mean (SD))	1111	2.34	(0.09)
Phosphate, mmol/L (n, mean (SD))	1116	1.10	(0.18)
CPP, mmol^2^/L^2^ (n, mean (SD))	1111	2.57	(0.43)
Vitamin D_2_ & D_3_, nmol/L (n, mean (SD))	1147	59.35	(24.00)
PTH, pmol/L (n, mean (SD))	1152	3.66	(1.97)
OPG, nmol/L (n, mean (SD))	1175	1.88	(0.59)
Troponine, ng/L (mean (SD))	1175	6.34	(24.78)
CRP, mg/L (n, mean (SD))	1179	2.89	(4.92)
SuPAR, ng/L (n, mean (SD))	1175	2.83	(0.97)
Creatinine, μmol/L (n, mean (SD))	1142	71.38	(16.87)
Cystatin C, mg/L (n, mean (SD))	1116	0.93	(0.17)
Urate, mmol/L (n, mean (SD))	1116	1.31	(32.71)
EGFR, mL/min (n, mean (SD))	1113	92.61	(12.11)
ABI (n, mean (SD))	1205	1.12	(0.13)
CAC score			
CAC = 0 (n (%))		666	(55.1 %)
CAC 1–99 (n (%))		364	(30.1 %)
CAC 100–399 (n (%))		112	(9.3 %)
CAC ≥400 (n (%))		66	(5.5 %)

### 3.2 Cardiovascular events

Out of the 1211 study participants, 92 (7.6 %) had a CV-event while 1059 (87.5 %) participants had no event. 10 participants (0.8 %) departed the country and 50 (4.1 %) died from other causes than CVD. The 92 CV-events were defined by first diagnosis and were 33 (35.9 %) strokes, 26 (28.3 %) MI's, 10 (10.9 %) heart failures, 18 (19.6 %) coronary artery revascularizations, and 5 (5.4 %) deaths from CVD (Table 2).

**Table 2:** Incidence of first cardiovascular event in a 10-year follow-up of the DanRisk study.

Status (n (%))	First registered event	
No event	1059	(87.5 %)
Departed	10	(0.8 %)
Death by other causes	50	(4.1 %)
Event	92	(7.6 %)
Stroke	33	(35.9 %)
Myocardial Infarction	26	(28.3 %)
Heart failure	10	(10.9 %)
Coronary Artery Revascularization	18	(19.6 %)
Cardiovascular death	5	(5.4 %)

### 3.3 Risk prediction of cardiovascular events

**Fig. 1:**
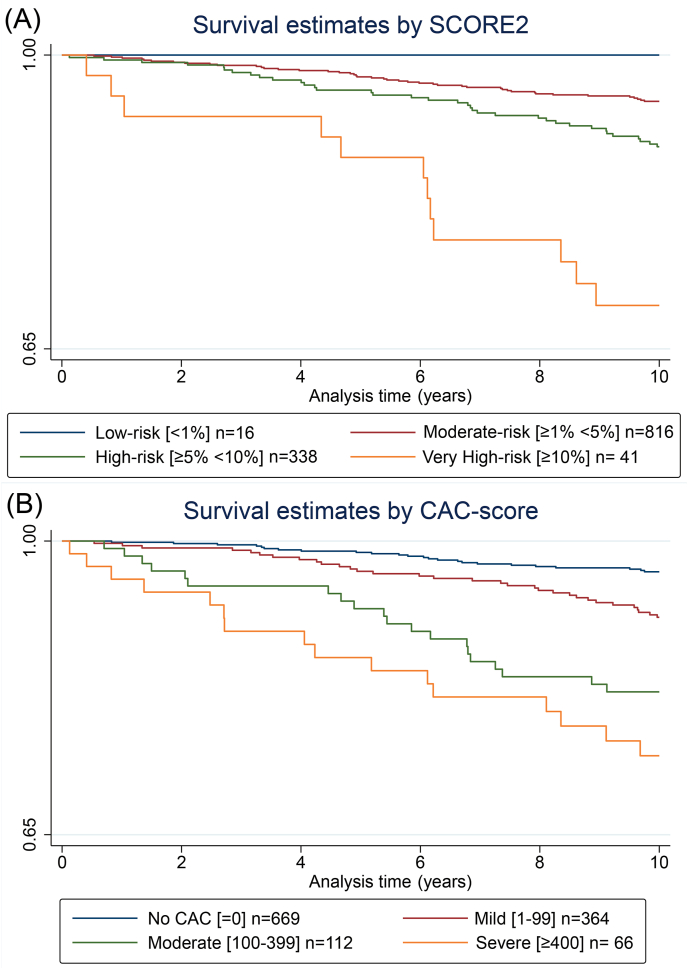
Kaplan Meier survival estimates curves of SCORE2 (A) and CAC-score (B) and cardiovascular events.

Survival estimates over time for SCORE2, and CAC-score are presented in Kaplan Meier diagrams in Fig. 1. Kaplan Meier curves for each biomarker are shown in Appendix 1. For SCORE2, 0 (0.0 %) of 16 participants in the low-risk group had an event, 44 (5.4 %) of 816 participants in the moderate risk group had an event, 36 (10.7 %) of 338 participants in high-risk group had an event and 12 (29.3 %) of 41 participants in the very-high risk group had an event. For CAC-score, 24 (3.6 %) of 666 participants with CAC-score 0 had an event, 32 (8.8 %) of 364 participants with CAC-score 1–99 had an event, 20 (17.9 %) of 112 participants with CAC-score 100–399 had an event and 16 (24.2 %) of 66 participants with CAC-score ≥400 had an event.

**Fig. 2:**
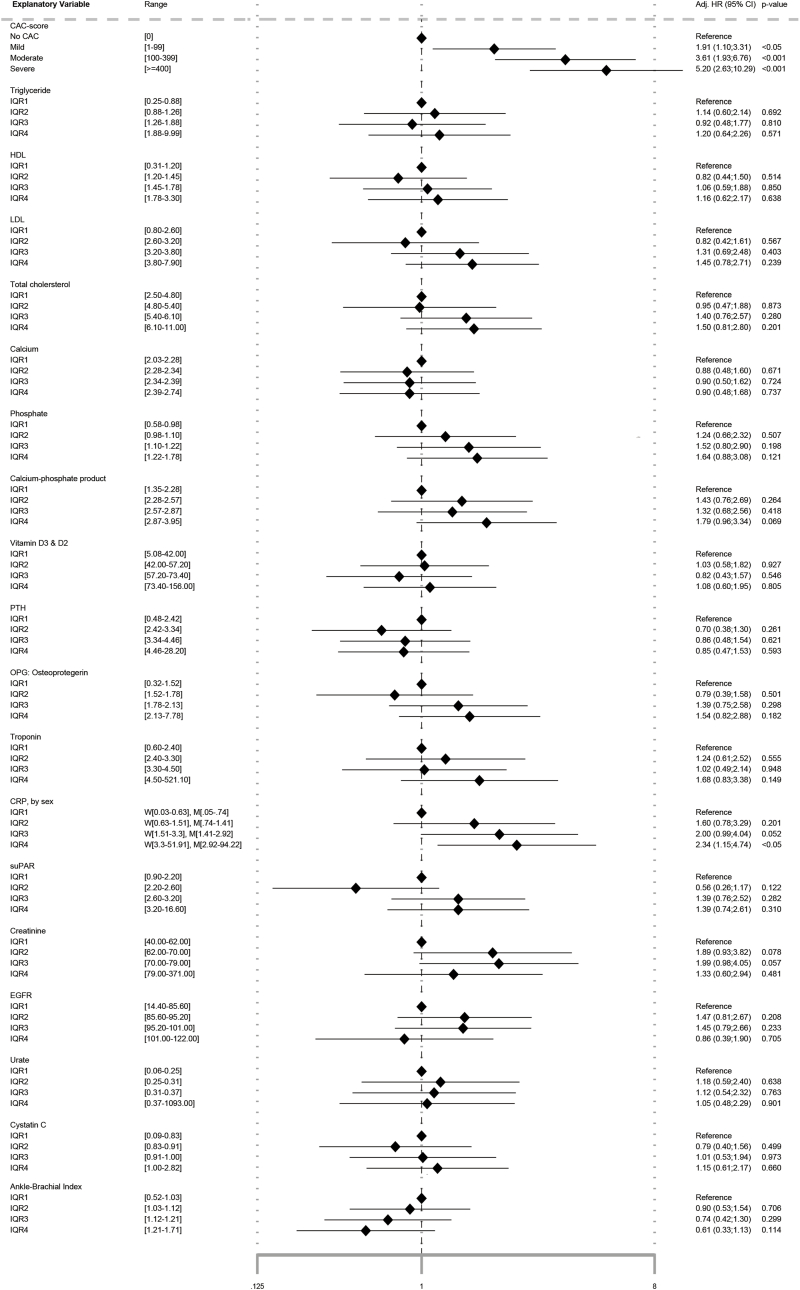
Forest Plot of adjusted∗ cox proportional hazard ratios (HR) in a 10-year survival analysis of CAC-score and biomarkers on cardiovascular events. ∗Adjusted for sex, age, body mass index, smoking status, hypertension, diabetes, hyperlipidemia, and family history of cardiovascular disease. CPP: calcium-phosphate product, CRP: C-reactive protein, EGFR: estimated glomerular filtration rate, HDL: high-density lipoprotein, LDL: low-density lipoprotein, OPG: osteoprotergerin, PTH: parathyroid hormone, SuPAR: soluble urokinase-type plasminogen activator receptor,.

Adjusted HR for each biomarker is presented in a Forest Plot in Fig. 2. The low-risk group according to SCORE2 had no events, and calculation of HR was therefore not possible. At a predefined 5 % significance level, CAC-score was significantly associated with CV-events with an HR of 1.9 (95%CI: 1.1; 3.3 – *p<*0.05), 3.6 (95%CI: 1.9; 6.8 – *p<*0.001), and 5.2 (95%CI: 2.6; 10.3 – *p<*0.001) for CAC-score 1–99, CAC-score 100–399 and CAC-score ≥400, respectively. HR for the highest quartile of CRP (women>3.3 and men>2.9) was 2.3 (95%CI: 1.2; 4.5). None of the remaining biomarkers (triglyceride, HDL, LDL, total cholesterol, calcium, phosphate, CPP, vitamin D2 and D3, PTH, OPG, troponin I, suPAR, creatinine, eGFR, cystatin C, urate, and ABI) improved HR.

### 3.4 AUC Comparison

**Fig. 3:**
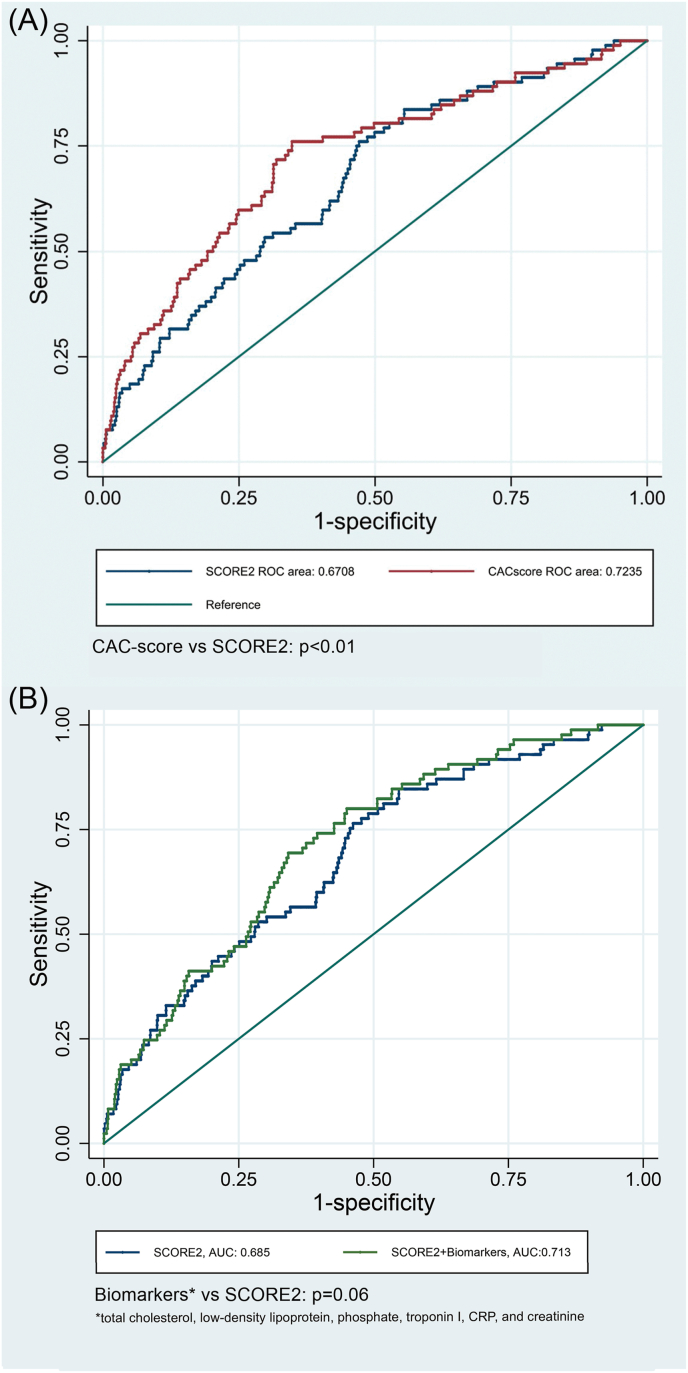
ROC-curves for SCORE2 and adjusted∗ CAC-score (A) and post-hoc combined biomarkers (B). ∗Adjusted for SCORE2.

ROC models with calculated AUC for SCORE2, CAC-score and 4 biomarker groups (1: lipids, 2: calcium-phosphate metabolism markers, 3: Ttroponin, inflammation markers and ABI, 4: markers of kidney function), are presented in Appendix 2.AUC for CAC-score, adjusted for SCORE2, were significantly higher when compared to the SCORE2-model (AUC_CAC_: 0.72, AUC_SCORE2_: 0.67, *p<*0.01). No individual biomarker adjusted for SCORE2 improved AUC compared to SCORE2. A combination of selected biomarkers borderline improved AUC (AUC_Biomarkers + SCORE2_: 0.71, AUC_SCORE2_: 0.67, *p=*0.06). ROC-models for CAC-score and the selected combined biomarkers are shown in Fig. 3.

### 3.5 NRI

Inclusion of CAC-score in the SCORE2 model improved the risk prediction with a NRI calculation of 63 % (*p<*0.0001). For participants with a CV-event, 73.9 % had increased probabilities and 26.1 % had decreased probabilities, compared to 42.4 % increased and 57.6 % decreased probabilities for participants without an event.

## 4 Discussion

### 4.1 Main findings

It is well known that CAC-score improves prediction of CV-events, when compared to the risk stratification algorithms, like SCORE and FRS. A new risk stratification algorithm called SCORE2 was introduced by the European Society of Cardiology (ESC) in 2021. In this prospective 10-year follow-up study, we confirmed that CAC-score improves risk stratification compared to SCORE2 in middle-aged men and women, with no previous CVD. Opposed to that, none of the evaluated biomarkers were able to improve the risk stratification for CV-events.

### 4.2 SCORE2 and CAC-score

According to SCORE2 guidelines [2], Denmark is classified as a low-risk European country, yet in this study of healthy middle-aged men and women, most participants were placed in the moderate and high-risk categories, and only 1.3 % and 3.4 % participants had a low or a very-high risk, respectively. The baseline risk estimation of the study participants when only using CAC-score was distributed differently from SCORE2. Half of the participants had no CAC and were in the lowest risk category, while 5.5 % had severe CAC. Number of events in the low-risk SCORE2 group compared to the no-CAC group were 0 (0.0 %) vs. 24 (3.6 %), respectively. On the other hand, there was almost an equal number of events for the very-high SCORE2 group and the severe CAC-group, 12 (29.3 %) vs. 16 (24.2 %), respectively.

The different numbers in the risk-groups for SCORE2 and CAC-score, as well as the large number but low percentage of events in the moderate and high-risk SCORE2 groups, might indicate that SCORE2 categorizes too many healthy people at a higher risk. Of importance, SCORE2 barely estimated any participants at low risk (1.3 %), which might lead to unnecessary anxiety. Overestimation of risk estimation is a well-known phenomenon in previous risk stratification models [10], and the result of this study suggests that this might be true, also for the SCORE2 model. The number of events in the no-CAC compared to the low-risk SCORE2 group might be surprising, as having no-CAC is considered having a low risk of CVD. It’s important to note, that in this study, participants have been screened using a non-contrast CT-scan, and we have therefore not been able to look at and determine the total plaque burden of both soft and hard plaques. The number of events could therefore be due to a non-detected soft plaque burden.

In this study, we confirmed that CAC-score improved risk prediction as HR’s increased with increasing CAC-score. Additionally, CAC-score improved AUC for SCORE2, also supporting that CAC-score improved the risk prediction compared to SCORE2 alone. Previous studies [[6], [7], [8],^11^] support the use of CAC-score for reclassification for those placed at intermediate risk. This current study also found support of including CAC-score in the SCORE2-model, as the NRI calculation showed an improvement for the risk estimation of 67.0 % participants (*p<*0.0001).

### 4.3 Biomarkers

The 2021 ESC guidelines on CVD prevention [4], do not recommend use of biomarkers, besides lipids, because of limited proof of their ability to improve risk stratification.

Total-, LDL- and HDL-cholesterol are included in the SCORE2 risk calculation because of their predictive ability and association with CVD found in previous research [2]. We were unable to confirm this association. This is probably due to our relatively small sample size, as the association is well-established in prior studies.

Of the biomarkers tested in the calcium-phosphate metabolism group, only phosphate showed to have a slight association with CV-events. Though our results were not significant, the association correlates with previous study findings for phosphate and risk of CVD [12]. Dhingra et al. [12] found a significant association between increased phosphate levels and risk of CVD, and our study had an almost equal HR for the highest IQR of phosphate compared to Dhingra et al. [12]. The latter study had a three times sample size and twice as many events, and accordingly a stronger power to detect the weak association between phosphate and CV-events.

This study was not able to find any significant association between calcium, phosphate, CPP, PTH or OPG and CV-events.

A meta-analysis by Fowkes et al. [13] found an the association between low ABI (≤0.90) and the 10-year risk of all-cause mortality, CV-mortality, and CV-events, even adjusted for FRS. Yeboah et al. [6] found similar results of an increased risk of CVD with decreased continuous values of ABI. Our study found decreasing HR with increasing ABI, and though the results were not significant the pattern supports the results of the Fowkes et al. [13] and Yeboah et al. [6] studies.

Opposed to several previous studies [[14], [15], [16]] we found no association between troponin I and the risk of CVD. In common, these three prior studies had a very large sample size, and with a huge number of participants they were able to find a small signal.

CRP was the only biomarker in our study to show any significance in risk prediction. The highest quartile of CRP had an over two times higher risk of CV-events. Our findings are in agreement with several prior studies [6,17,18]. SuPAR is often associated together with CRP as a predictor of CVD. Eugen-Olsen et al. [19] found an association between elevated SuPAR-levels and increased risk of CVD. Furthermore, a previous DanRisk substudy, Diederichsen et al. [18], looked at both CRP and SuPAR, and found both biomarkers as individual predictors of CVD. Despite the extended follow-up in this DanRisk substudy, we were unable to confirm the association between SuPAR and CV-events.

Markers of kidney function have previously been linked to CVD [4,[20], [21], [22]]. Specifically, eGFR is often mentioned in relation to chronic kidney disease (CKD) and risk of CVD. In the ESC guidelines [4] it is discussed that patients with CKD (eGFR<60 ml/min) have an increasing risk of CVD with decreasing values of eGFR. Our study results are difficult to compare to these findings, as our study did not adjust for kidney function, and the guidelines indicate that the association is seen in patients with diagnosed CKD. The vast majority of our study participants did not have CKD, and this may explain why we did not find any association between markers of kidney function and risk of CV-events.

Finally, we post-hoc selected biomarkers proven to be associated with CVD in previous studies. These include total cholesterol, low-density lipoprotein, phosphate, troponin I, CRP, and creatinine, and were combined in supplementary AUC calculation. The combination of these biomarkers was able to show a borderline significant improvement of AUC compared to SCORE2). Even in combination, these biomarkers are not comparable with CAC-score.

### 4.4 Study strengths and limitations

These results are based on a random selection of healthy middle-aged Danes. A strength is our equal distribution of men and women, as well as 50- and 60-year-olds, reducing bias and increasing generalizability in terms of gender and age. Since the participants were mainly Caucasian our study results may not be comparable to other ethnicities. The use of the Danish registers for follow-up is also a strength, as we were able to follow up on the vast majority of study participants, minimizing the risk of recall and non-response bias. Although we have a relatively long follow-up, the sample size is quite small to detect weak associations. Register-based data has its limitations, as the quality of some data may be questionable. Especially the validity of the Death-register can be questioned.

The use of biomarkers in a long cohort study has limitations as biomarkers often only represent a certain moment in time, and many factors can influence the levels of these biomarkers. Some can change daily, like inflammation markers, and others can be elevated due to other underlying reasons. Our study was also not able to include every biomarker, but inclusion of other biomarkers like pro-brain natriuretic peptide (ProBNP), which is acknowledged as a good risk-predictor, should be considered. Unfortunately, ProBNP was not available at the start of this study.

## 5 Conclusion

In this study, we confirmed the importance of CAC-scores to improve the risk stratification even when using the new SCORE2. Unlike several prior very large studies, who found an association between several biomarkers and CVD, our study was unable to show a statistic significant association between 19 biomarkers and CV-events or improvement of the risk stratification.

## Conflict of interest

The authors declared no potential conflicts of interest with respect to the research, authorship and/or publication of this article.

## Financial support

This work was supported by 10.13039/501100009708Novo Nordisk Fonden.

## Data availability

Individual participants’ data cannot be shared, as all outcome analysis are performed on servers in the Danish Health Data Authority Registry.

## Authors’ contributions

MS, MHF, JHR and ACPD conceived and designed the study. FHS, JL, NPRS, ACPD, LMR played key roles in recruitment of participants. MS, JHR, SH, FNJ, FHS, JL, NPRS, LMR and ACPD contributed to the acquisition, analysis, or interpretation of data for the work. MS and ACPD drafted the report.

All authors provided critical input into revised versions of the manuscript. All gave final approval and agreed to be accountable for all aspects of work ensuring integrity and accuracy.

## Declaration of competing interest

The authors declare that they have no known competing financial interests or personal relationships that could have appeared to influence the work reported in this paper.
