# Supplementary material for: Coronary artery calcification score and 19 biomarkers on cardiovascular events; a 10-year follow-up DanRisk substudy
Source: Atheroscler Plus. 2024 Sep 24;58:9–15. doi: 10.1016/j.athplu.2024.09.003 (PMC11470180; doi:10.1016/j.athplu.2024.09.003)
Supplement: Multimedia component 1 [file mmc1.docx]

# Appendix 1 - Biomarker Kaplan Meier Survival Curves.

##
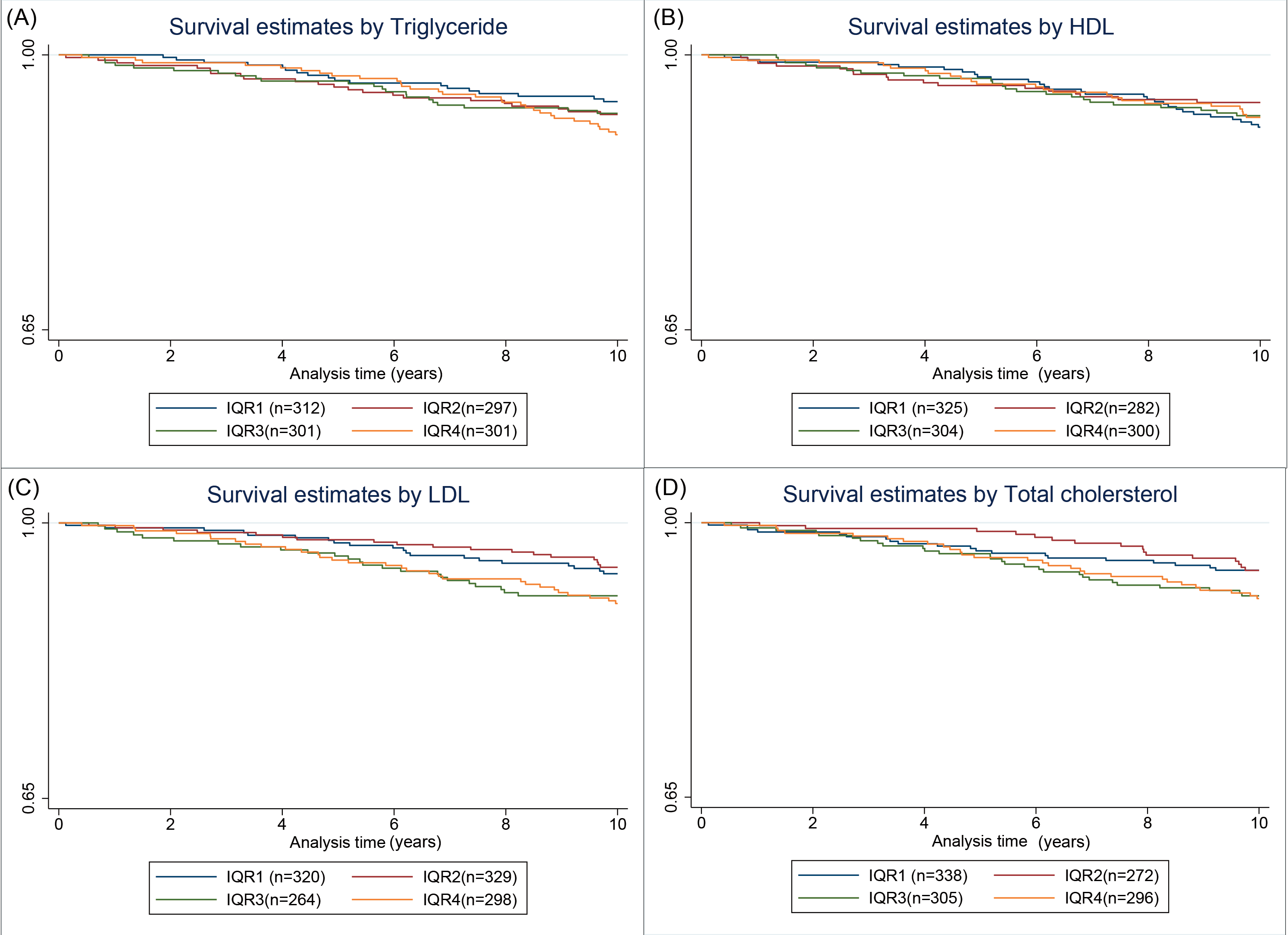
Lipid markers

##
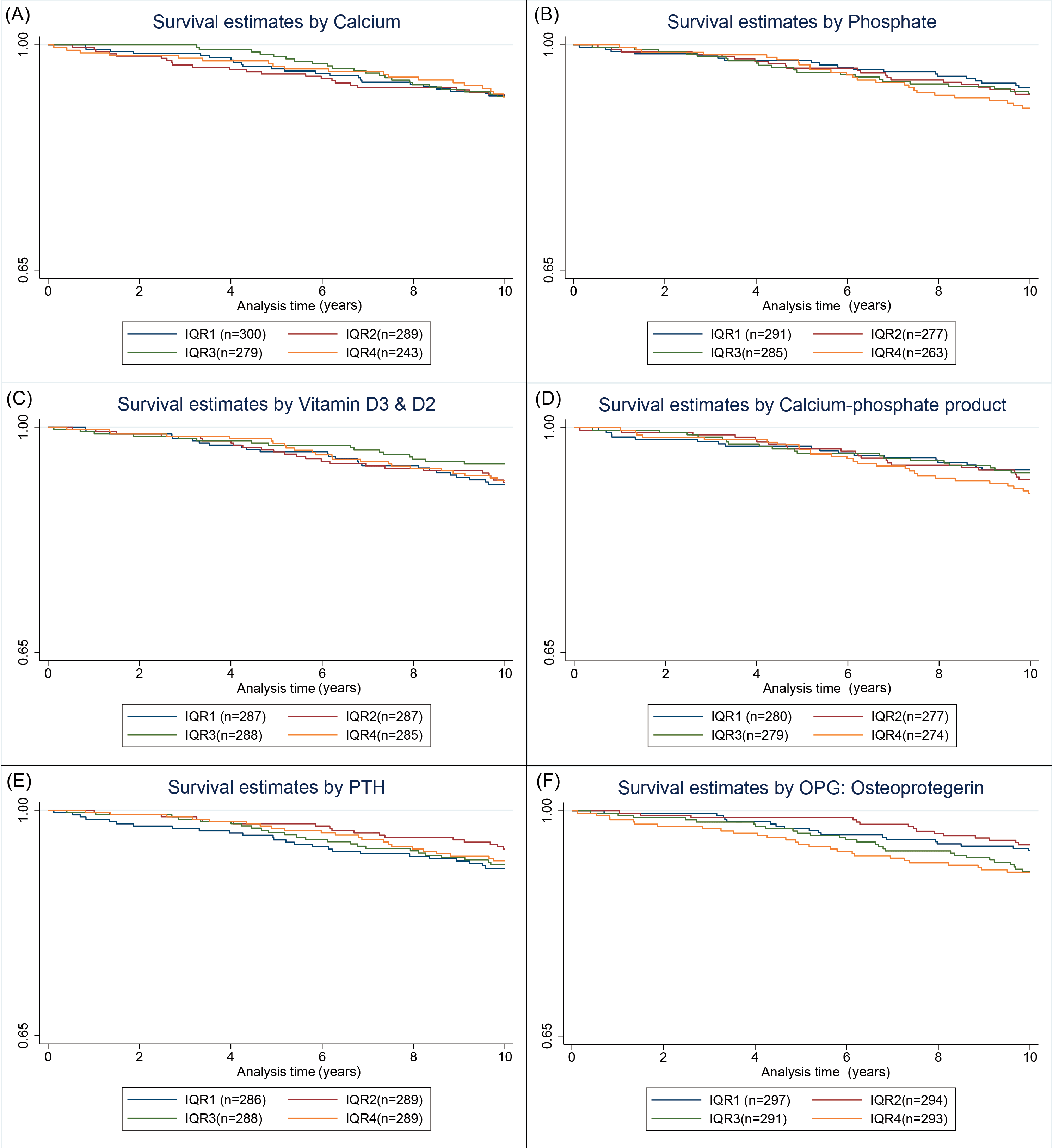
Calcium-phosphate metabolism markers

##
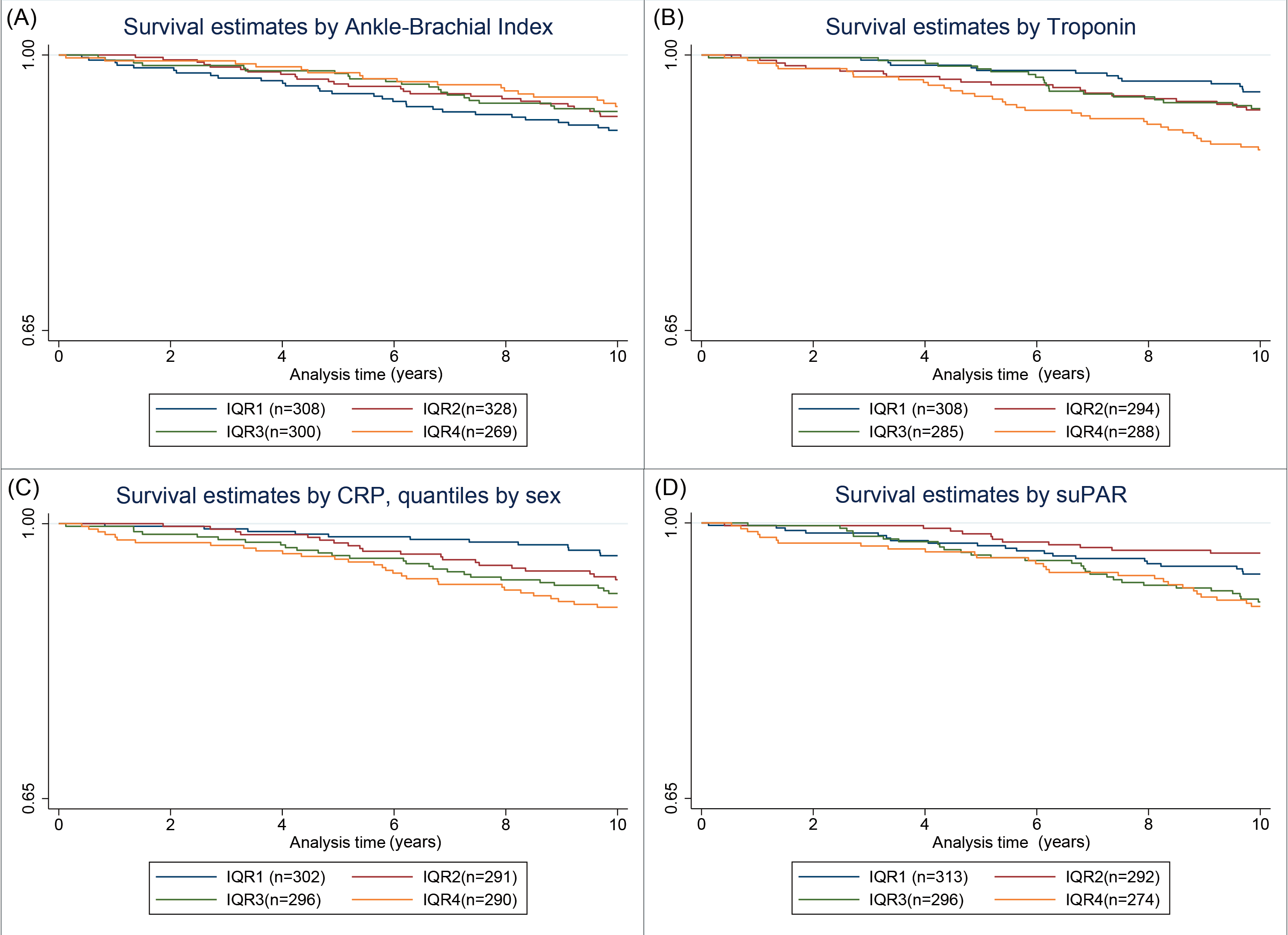
ABI, troponin I and inflammation markers

##
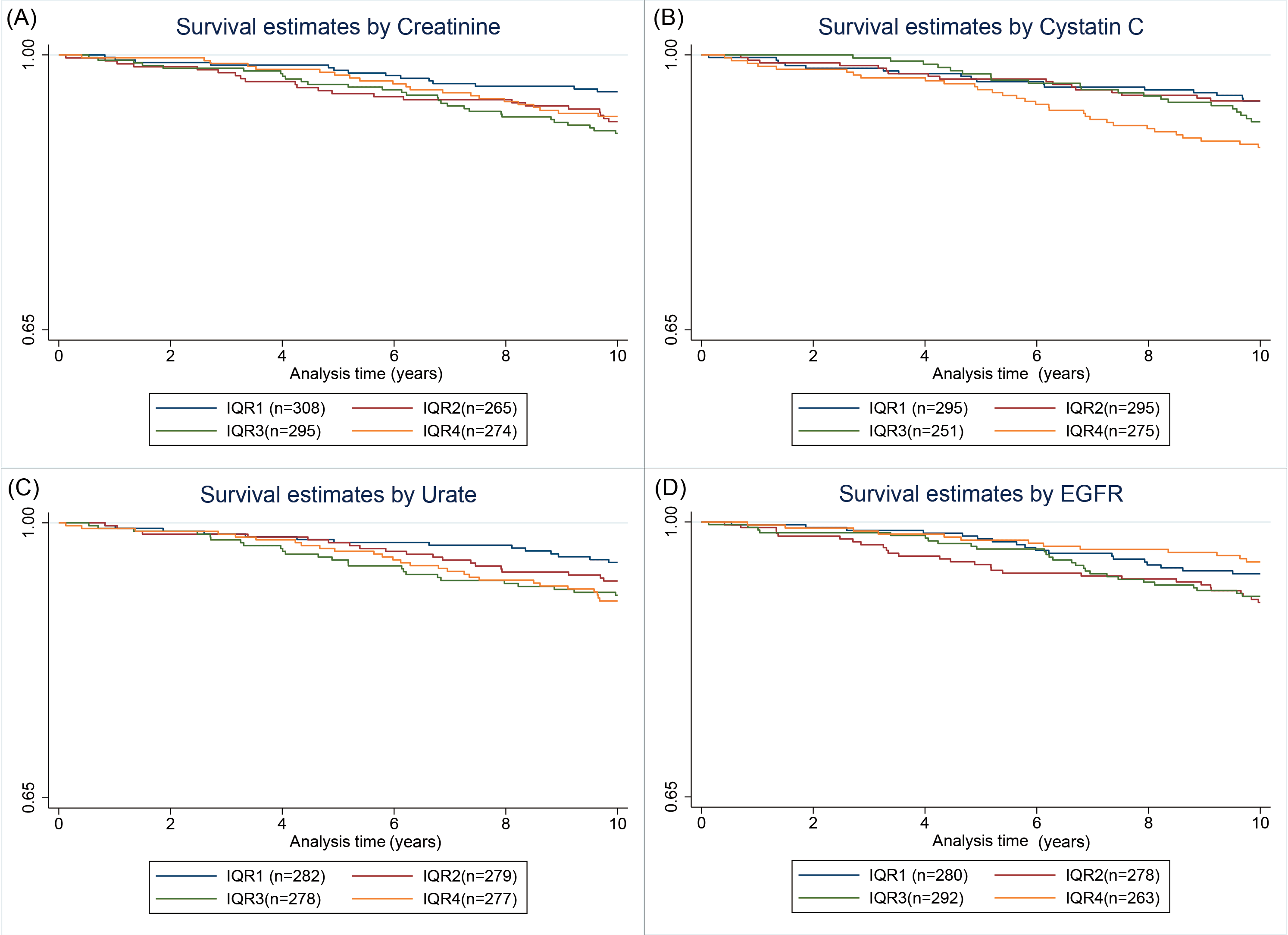
Markers of kidney function

# Appendix 2 – ROC-curves

ROC for SCORE2, adjusted* CAC-score and biomarkers: (A) Lipids, (B) Calciumphosphate metabolism, (C) ABI, Troponine I and inflammation markers and (D) Markers of kidney funcion


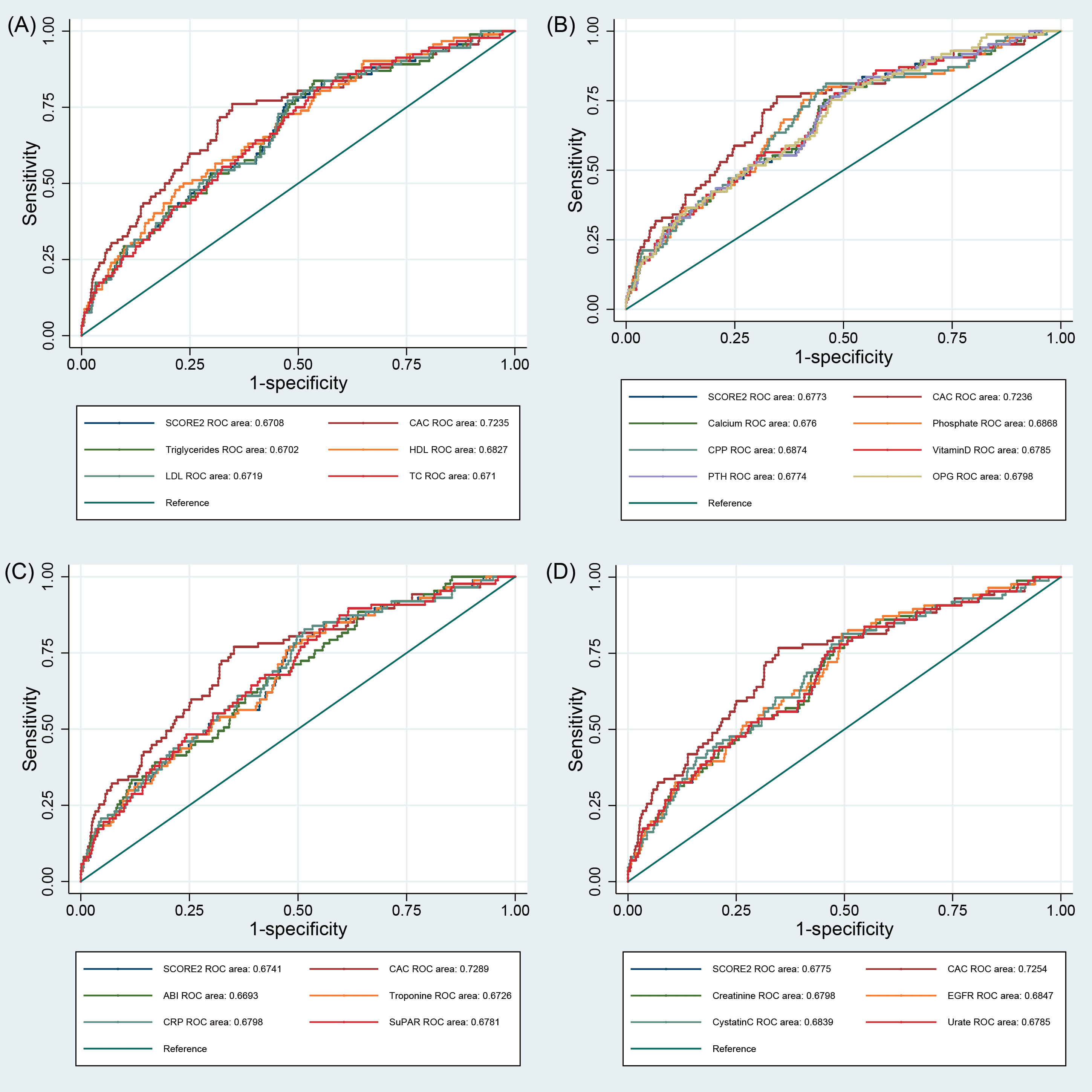
* Adjusted for SCORE2
